# The Membrane Composition Defines the Spatial Organization and Function of a Major Acinetobacter baumannii Drug Efflux System

**DOI:** 10.1128/mBio.01070-21

**Published:** 2021-06-17

**Authors:** Maoge Zang, Hugo MacDermott-Opeskin, Felise G. Adams, Varsha Naidu, Jack K. Waters, Ashley B. Carey, Alex Ashenden, Kimberley T. McLean, Erin B. Brazel, Jhih-Hang Jiang, Alessandra Panizza, Claudia Trappetti, James C. Paton, Anton Y. Peleg, Ingo Köper, Ian T. Paulsen, Karl A. Hassan, Megan L. O’Mara, Bart A. Eijkelkamp

**Affiliations:** a Molecular Sciences and Technology, College of Science and Engineering, Flinders University, Adelaide, South Australia, Australia; b Research School of Chemistry, College of Science, The Australian National University, Canberra, Australian Capital Territory, Australia; c Department of Molecular Sciences, Macquarie University, Sydney, New South Wales, Australia; d Flinders Centre for Nanoscale Science and Technology, Flinders University, Adelaide, South Australia, Australia; e Research Centre for Infectious Diseases, School of Biological Sciences, University of Adelaide, Adelaide, South Australia, Australia; f Infection and Immunity Program, Monash Biomedicine Discovery Institute and Department of Microbiology, Monash University, Clayton, Victoria, Australia; g Department of Infectious Diseases, The Alfred Hospital and Central Clinical School, Monash University, Melbourne, Victoria, Australia; h School of Environmental and Life Sciences, University of Newcastle, Callaghan, New South Wales, Australia; Louis Stokes Veterans Affairs Medical Center

**Keywords:** bacterial, host lipids, antibiotics, resistance, RND efflux

## Abstract

Acinetobacter baumannii is one of the world’s most problematic nosocomial pathogens. The combination of its intrinsic resistance and ability to acquire resistance markers allow this organism to adjust to antibiotic treatment. Despite being the primary barrier against antibiotic stress, our understanding of the A. baumannii membrane composition and its impact on resistance remains limited. In this study, we explored how the incorporation of host-derived polyunsaturated fatty acids (PUFAs) is associated with increased antibiotic susceptibility. Functional analyses of primary A. baumannii efflux systems indicated that AdeB-mediated antibiotic resistance was impacted by PUFA treatment. Molecular dynamics simulations of AdeB identified a specific morphological disruption of AdeB when positioned in the PUFA-enriched membrane. Collectively, we have shown that PUFAs can impact antibiotic efficacy via a vital relationship with antibiotic efflux pumps. Furthermore, this work has revealed that A. baumannii’s unconditional desire for fatty acids may present a possible weakness in its multidrug resistance capacity.

## OBSERVATION

**FIG 1:**
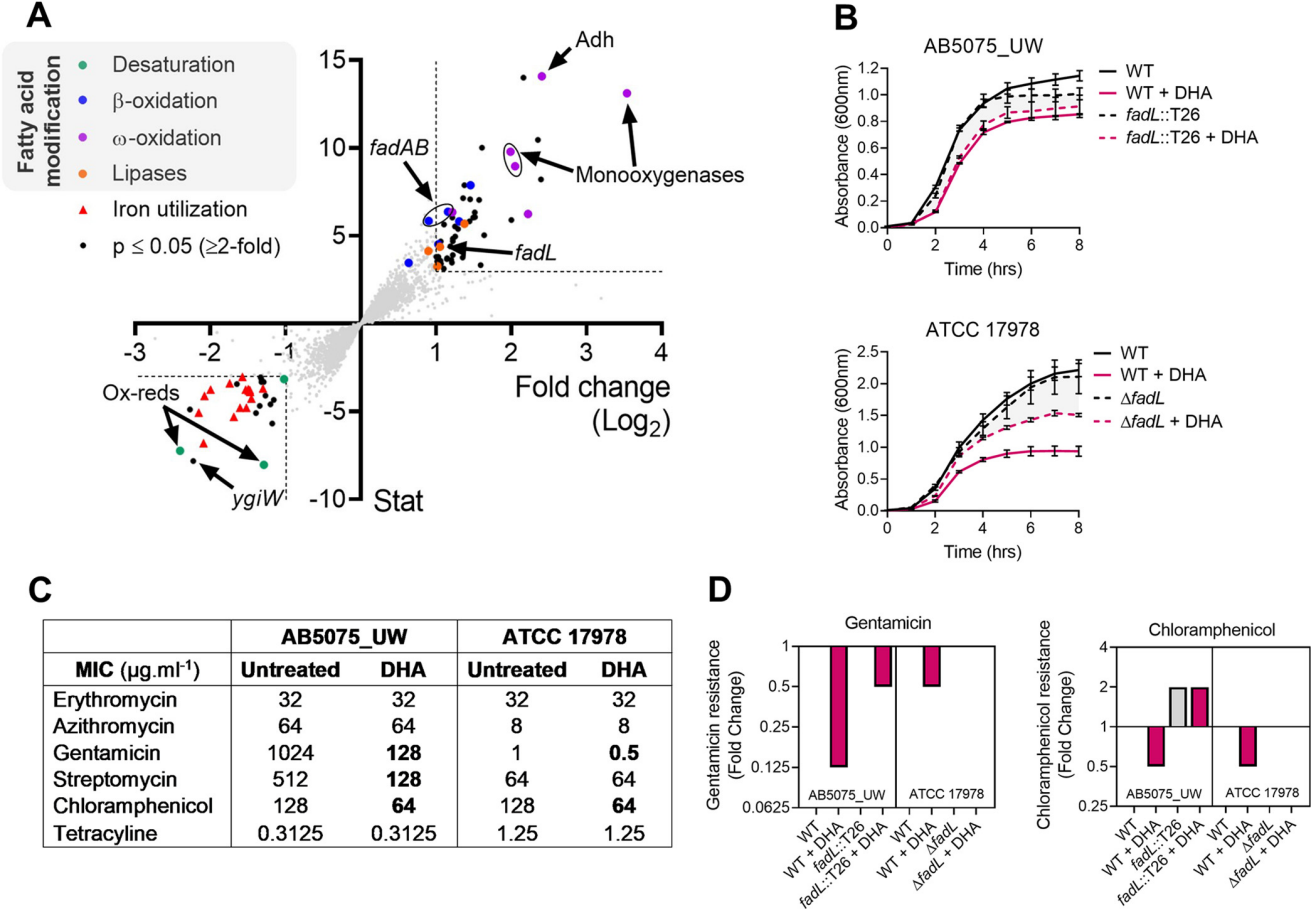
Transcriptomic and resistance analyses of A. baumannii under DHA stress. (A) A. baumannii strain AB5075_UW was exposed to 0.25 mM DHA for 30 min. The transcriptomes of untreated and treated cells were examined in biological triplicates by Illumina sequencing. The log_2_ fold change in transcription of each gene of the genome in response to DHA stress is presented on the *x* axis and the statistical evaluation on the *y* axis. Differentially expressed genes involved in similar biological processes are indicated in distinct colors: fatty acid desaturation in green (e.g., desaturases and oxidoreductases), fatty acid β-oxidation in blue (e.g., *fadAB* and *fadL*), fatty acid ω-oxidation in purple (e.g., alcohol dehydrogenases [Adh] and monooxygenases), lipases in orange, and genes with putative roles in iron acquisition in red. The data represent 3 biological replicates. (B) The growth (absorbance at 600 nm) of A. baumannii AB5075_UW and ATCC 17978 wild-type (WT) and *fadL* mutant strains was examined with and without 0.25 mM DHA. Data represent the mean (±standard error of the mean [SEM]) from biological quadruplicates. (C) The MIC was defined for a range of common antibiotics in AB5075_UW and ATCC 17978 with and without 0.25 mM DHA. The data are the mode of biological triplicates. (D) The fold change in the MIC (relative to untreated WT cells for gentamicin and chloramphenicol was examined in the AB5075_UW (*fadL*::T26) and ATCC 17978 (Δ*fadL*) backgrounds. The gray bars indicate untreated samples, and those in burgundy represent samples treated with 0.25 mM DHA. The data represent a value of 1 (i.e., no change) in cases where no bar is visible. The data are the mode from biological triplicates.

Acinetobacter baumannii is one of the world’s most notorious multidrug resistant pathogens (1, 2), yet how it responds to host-mediated stress is poorly understood. Previous reports have shown that this human pathogen displays susceptibly to host-derived omega-3 polyunsaturated fatty acids (PUFAs), such as docosahexaenoic acid (DHA) (3, 4). Here, we examined the transcriptomic responses to 0.25 mM DHA stress to identify possible genetic traits responsible for these outcomes. Although plasma DHA concentrations range from 0.1 to 0.2 mM in humans on a typical Western diet (5, 6), this can increase to levels greater than 0.4 mM DHA in populations with higher marine fish oil intake (7). Differential expression analyses revealed transcripts for 53 and 32 genes to be of higher and lower abundance, respectively (≥2-fold change, *P ≤ *0.05), in DHA treated compared to untreated A. baumannii AB5075_UW cells (see Table S1 in the supplemental material). Among the most downregulated genes were those coding for two putative oxidoreductases, ABUW_3843 (5.29-fold) and ABUW_1104 (2.44-fold) (Fig. 1A; see Fig. S1A to C in the supplemental material), which are likely to assist in the electron transport required for their cotranscribed fatty acid desaturases. This is likely to be a specific response to restrict the introduction of more double bonds in acyl chains following PUFA treatment. Consistently, an increase in DHA susceptibility in the repressor mutant of this cluster was observed (Fig. S1D). Despite minimizing the exposure to DHA, the transcriptomic analyses also revealed potential general stress responses. A homologue of the Escherichia coli stress tolerance gene *ygiW* was significantly downregulated 4.68-fold. Further, a total of 18 genes associated with iron utilization were significantly downregulated (Fig. 1A), which could not be linked directly to fatty acid homeostasis. A number of genes with putative roles in ω-oxidation were found to be significantly upregulated upon DHA treatment (Fig. 1A). The resulting product of the ω-oxidation pathway, fatty dicarboxylic acids, can be catabolized by β-oxidation or dicarboxylate catabolic (*dca*) pathways (8), and several putative components in both these pathways were upregulated under DHA stress. A predicted long-chain fatty acid transporter gene (*fadL*; ABUW_0724) was among the upregulated genes of the β-oxidation pathway. Importantly, *fadL* mutants in either the A. baumannii AB5075_UW or ATCC 17978 background displayed enhanced tolerance to DHA stress (Fig. 1B; Fig. S1). Although transcriptomic profiling showed the downregulation of two desaturases to restrict introduction of double bonds following DHA treatment, it also revealed the upregulation of *fadL*. This somewhat greedy and unconditional acquisition of energy-rich DHA has detrimental impacts on its fitness. Unlike A. baumannii, which can be represented in a vast array of environmental habitats, Streptococcus pneumoniae is fully host adapted and is attuned to the diligent acquisition of distinct fatty acids in the host environment, which is facilitated by the concerted action of having selective proteins (FakB1, saturated FAs; FakB2 monounsaturated FAs; FakB3, PUFAs), as well as appropriate transcriptional regulation of these systems (9, 10).

10.1128/mBio.01070-21.2 FIG S1 RNAseq and qRT-PCR analyses of lipid homeostasis genes. (A) The transcriptional changes following DHA treatment (0.25 mM for 30 min) were analyzed by transcriptome sequencing (RNA-seq [gray]) and qRT-PCR (purple) and are displayed as times fold difference compared to untreated (UT) cells. (B and C) The RNA-seq transcript coverage (*y* axis) for UT (gray) and DHA-treated (burgundy) cells is displayed for two putative gene clusters that enable fatty acid desaturation ABUW_3842 to -3844 (B) and ABUW_1103 to -1105 (C). (D) The difference in the OD_600_ between DHA-treated (0.25 mM) and UT cells was determined after 3 h of growth in LB medium. The data represents the mean from 3 (±SEM) biological replicates. Statistical analyses (all compared to the data obtained for strain AB5075_UW) were performed using an ANOVA (*, *P* < 0.05; ***, *P* < 0.001). Download FIG S1, TIF file, 0.7 MB. Copyright © 2021 Zang et al. 2021 Zang et al. https://creativecommons.org/licenses/by/4.0/ This content is distributed under the terms of the Creative Commons Attribution 4.0 International license.

10.1128/mBio.01070-21.4 TABLE S1 Transcriptomic responses to DHA stress. Download Table S1, DOCX file, 0.03 MB. Copyright © 2021 Zang et al. 2021 Zang et al. https://creativecommons.org/licenses/by/4.0/ This content is distributed under the terms of the Creative Commons Attribution 4.0 International license.

Considering PUFA incorporation affects A. baumannii membrane permeability (4), we examined antimicrobial susceptibility of A. baumannii strains AB5075_UW and ATCC 17978 with or without DHA supplementation (Fig. 1C). The presence of subinhibitory amounts of DHA resulted in a 4- to 8-fold decrease in aminoglycoside resistance (gentamicin and streptomycin, respectively) and 2-fold decrease in chloramphenicol resistance in strain AB5075_UW. These differences were less dramatic in the more-antibiotic-susceptible strain ATCC 17978, where only 2-fold reductions were observed for gentamicin and chloramphenicol following cotreatment with DHA. Although commonly known for their ability to interact with phospholipids, resistance to macrolides (erythromycin and azithromycin) was not affected by PUFA treatment. In addition to altering antibiotic resistance, we examined the impact of PUFA supplementation on oxidative stress tolerance in strain AB5075_UW and revealed that PUFA treatment impacted tolerance to paraquat, which induces the formation of intracellular superoxide stress (see Fig. S2 in the supplemental material). Contrastingly, PUFA-treated bacteria were not more susceptible to exogenously supplemented hydrogen peroxide compared to untreated bacteria (Fig. S2). Examination of the impact of DHA upon gentamicin or chloramphenicol susceptibility in a *fadL* mutant in either an AB5075_UW or ATCC 17978 background revealed that increased susceptibility to these compounds occurs primarily following DHA uptake into the cell (Fig. 1D). In contrast to A. baumannii, no dramatic changes (≤2-fold) in antibiotic susceptibility following DHA treatment were seen in the Gram-positive bacterium, Streptococcus pneumoniae (see Table S2 in the supplemental material). Hence, our study supports the identification of a plausible pathogen-specific Achilles’ heel, this being the active acquisition of DHA by A. baumannii and the subsequent increase in antibiotic susceptibility.

10.1128/mBio.01070-21.3 FIG S2 Oxidative stress tolerance in PUFA-treated cells. The effects of 160 μM H_2_O_2_ or 40 μM paraquat on A. baumannii AB5075_UW grown in LB broth with or without 125 μM DHA were quantified by comparing the times for cultures to reach 50% of the maximum growth. This was calculated from OD_600_ measurements taken every 30 min. Data represent the mean (±SEM) of the growth delays from at least 5 biological replicates. ****, *P* < 0.0001. Download FIG S2, TIF file, 0.1 MB. Copyright © 2021 Zang et al. 2021 Zang et al. https://creativecommons.org/licenses/by/4.0/ This content is distributed under the terms of the Creative Commons Attribution 4.0 International license.

10.1128/mBio.01070-21.5 TABLE S2 Minimal inhibitory concentration of Streptococcus pneumoniae with and without DHA treatment. Download Table S2, DOCX file, 0.02 MB. Copyright © 2021 Zang et al. 2021 Zang et al. https://creativecommons.org/licenses/by/4.0/ This content is distributed under the terms of the Creative Commons Attribution 4.0 International license.

**FIG 2:**
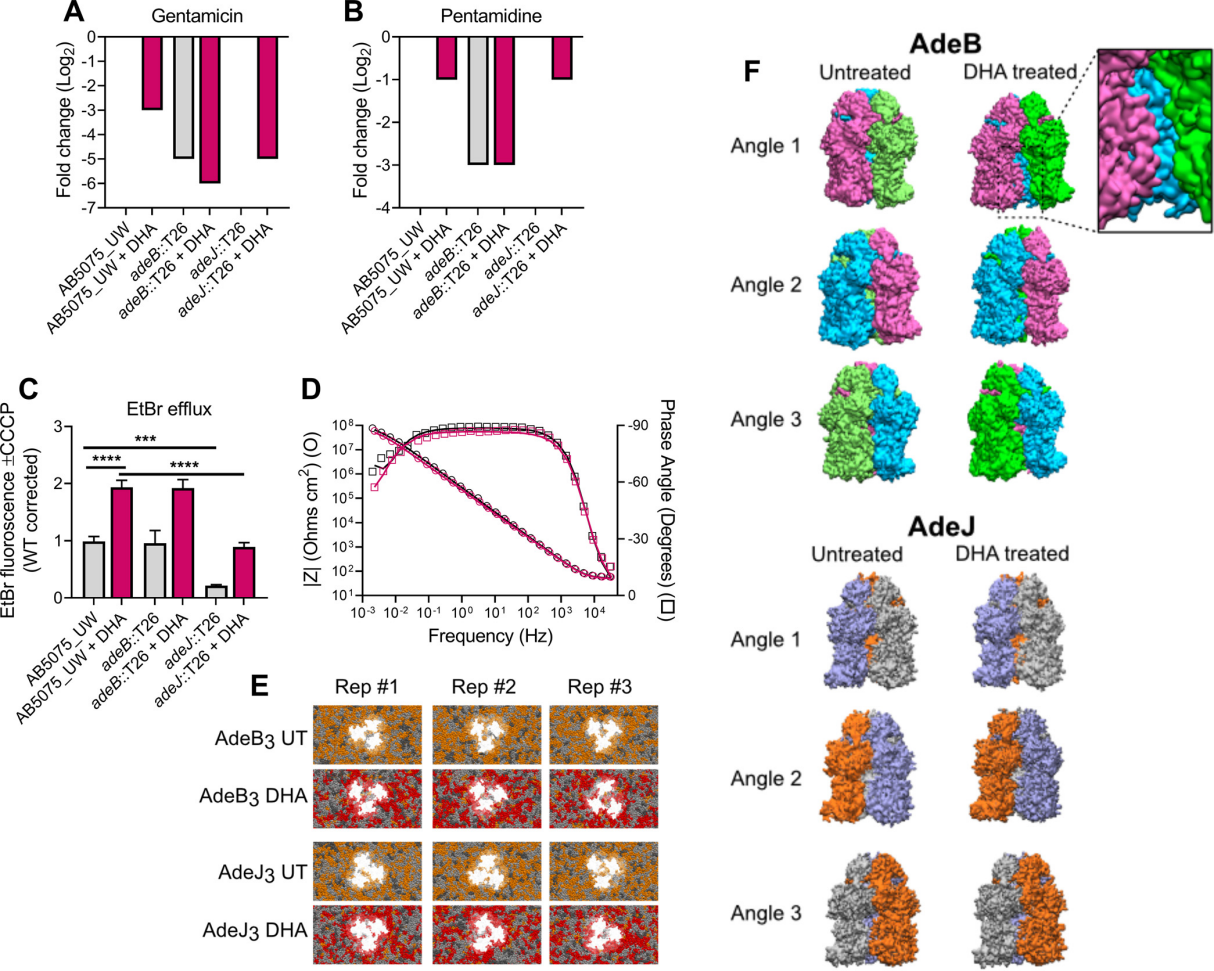
The interdependence of lipid homeostasis and RND efflux in A. baumannii. Fold changes in the MIC of gentamicin (A) or pentamidine (B) in AB5075_UW, *adeB*::T26, and *adeJ*::T26 strains, with or without DHA supplementation (250 μM), are shown. The data (mode) are representative of 6 biological replicates. The gray bars indicate untreated samples, and those in burgundy represent samples treated with DHA. The data represent no change from the untreated wild-type cells in cases where no bar is visible. (C) Accumulation of ethidium bromide (5 μM) in strain AB5075_UW and the *adeB*::T26 and *adeJ*::T26 strains, with or without 125 μM DHA. The efflux potential was defined by determining the difference between efflux-negative cells (40 μM CCCP) and actively effluxing cells (no CCCP). The data are representative of 8 or 4 biological replicates for the wild-type and the two mutants, respectively (±SEM). Statistical analyses were performed by analysis of variance (ANOVA) (***, *P* < 0.001; ****, *P* < 0.0001). (D) Bode plot of an untreated (UT [black]) and DHA-treated (purple) tBLM after formation. Symbols represent measured data (representative of 4 replicates), and solid lines represent a fit to an equivalent circuit of resistors and capacitors. (E) Snapshots from the final 5 μs of AdeB and AdeJ trimer simulations in the untreated and DHA-treated membranes. MD simulations were performed in triplicates (Rep #1, Rep #2, and Rep #3). Saturated lipids are shown in dark gray, monounsaturated lipids in light gray, diunsaturated lipids in orange, and PUFA-containing lipids in red. (F) Representative snapshots of AdeB and AdeJ trimer conformation from the final 5 μs of simulations in the untreated and DHA-treated membranes. Each protomer is colored differently to aid visualization of protomer interactions from each angle.

Considering the roles of resistance-nodulation-cell division (RND) efflux systems in lipid homeostasis and DHA resistance (4, 11, 12), we studied the efflux activities of AdeB and AdeJ in A. baumannii with and without omega-3 PUFA enrichment. We found that the specific roles of AdeB in gentamicin and pentamidine resistance were impacted by DHA treatment, as the resistance decreased to a greater extent when AdeB was present (i.e., in wild-type or *adeJ*::T26 cells) than in the *adeB*::T26 mutant (Fig. 2A and B). To delineate the relative impact of DHA on AdeJ efflux activity, we examined the ethidium bromide (EtBr) efflux potential, by analyzing the cells with and without treatment with the protonophore carbonyl cyanide *m*-chlorophenyl hydrazine (CCCP), which can indirectly prevent efflux from RND pumps by collapsing the proton motive force. We found that active efflux by AdeJ is required for preventing the accumulation of EtBr, but DHA did not negatively impact this process (Fig. 2C). Instead, the increased membrane permeability as a result of DHA incorporation required greater EtBr efflux, which was reflected in the enhanced EtBr efflux potential of DHA-treated cells.

Since RND efflux relies upon the proton motive force across the cytoplasmic membrane, we ascertained the possibility of ion leakage in the A. baumannii membrane following DHA incorporation. Lipid samples extracted from actively growing A. baumannii cells were used to generate tethered bilayer lipid membranes (tBLMs). Both tBLMs displayed typical electrochemical properties (Fig. 2D) and similar responses to the incorporation of the ion carrier valinomycin (see Table S3 in the supplemental material). These analyses illustrate that the observed dysfunction of AdeB is not a result of the membrane being compromised in its ability to retain a proton motive force following the incorporation of DHA.

10.1128/mBio.01070-21.6 TABLE S3 Electrochemical properties of tethered bilayer lipid membranes. Download Table S3, DOCX file, 0.02 MB. Copyright © 2021 Zang et al. 2021 Zang et al. https://creativecommons.org/licenses/by/4.0/ This content is distributed under the terms of the Creative Commons Attribution 4.0 International license.

Evidence is emerging that phospholipids can influence folding, structure, and function of some membrane proteins, including bacterial RND systems (13–15). To identify possible differences in AdeB and AdeJ conformations in the membrane, we studied the dynamics of AdeB and AdeJ (both modeled on the AdeB cryo-electron microscopy [cryo-EM] structure; 6OWS) in the A. baumannii phospholipid environment with and without omega-3 PUFA enrichment in coarse-grained molecular dynamics (MD) simulations. After 15 μs replicate simulations, the lipid annulus surrounding both AdeB and AdeJ was enriched with unsaturated lipids in the untreated A. baumannii membrane (Fig. 2E). PUFA-containing phospholipids were heavily localized around AdeB and AdeJ when the protein complexes were embedded in a DHA-treated A. baumannii membrane (Fig. 2E). Although the conformation of the AdeJ trimer was largely unaffected by these changes in its phospholipid environment, the conformation of AdeB displayed a dramatic shift, with the complete loss of the protein-protein interface between adjacent transmembrane domains of two AdeB protomers (Fig. 2F). When assessed in conjunction with functional assays of AdeB activity in the presence of DHA, these changes suggest a possible mechanism for disruption of the conformational cycling of AdeB required for efflux activity. Consistently, previous reports on the membrane-disrupting biocides on AdeABC have also linked a role in cell envelope integrity and AdeABC efflux activity (16). Overall, the data presented here provide insights into the interplay between the membrane lipid composition and specific RND efflux activities. Hence, our work has established a molecular basis for how the lipid bilayer composition and its biophysical properties may affect antibiotic treatment success.

Overall, this study has presented a comprehensive analysis of the antimicrobial effects of host fatty acids upon A. baumannii membrane biology. Although, omega-3 PUFA supplementation is unlikely to affect healthy individuals, those that are at increased risk of contracting bacterial infections may benefit, in particular during antibiotic treatment.

10.1128/mBio.01070-21.1 TEXT S1 Supplemental methods. Download Text S1, DOCX file, 0.1 MB. Copyright © 2021 Zang et al. 2021 Zang et al. https://creativecommons.org/licenses/by/4.0/ This content is distributed under the terms of the Creative Commons Attribution 4.0 International license.

10.1128/mBio.01070-21.7 TABLE S4 Bacterial strains included in the study. Download Table S4, DOCX file, 0.02 MB. Copyright © 2021 Zang et al. 2021 Zang et al. https://creativecommons.org/licenses/by/4.0/ This content is distributed under the terms of the Creative Commons Attribution 4.0 International license.

10.1128/mBio.01070-21.8 TABLE S5 Oligonucleotides included in the study. Download Table S5, DOCX file, 0.02 MB. Copyright © 2021 Zang et al. 2021 Zang et al. https://creativecommons.org/licenses/by/4.0/ This content is distributed under the terms of the Creative Commons Attribution 4.0 International license.

10.1128/mBio.01070-21.9 TABLE S6 Phospholipid species included in membrane modeling. Download Table S6, DOCX file, 0.03 MB. Copyright © 2021 Zang et al. 2021 Zang et al. https://creativecommons.org/licenses/by/4.0/ This content is distributed under the terms of the Creative Commons Attribution 4.0 International license.
