# Supplementary figures and images for: The Membrane Composition Defines the Spatial Organization and Function of a Major Acinetobacter baumannii Drug Efflux System
Source: mBio. 2021 Jun 17;12(3):e01070-21. doi: 10.1128/mBio.01070-21 (PMC8262998; doi:10.1128/mBio.01070-21)

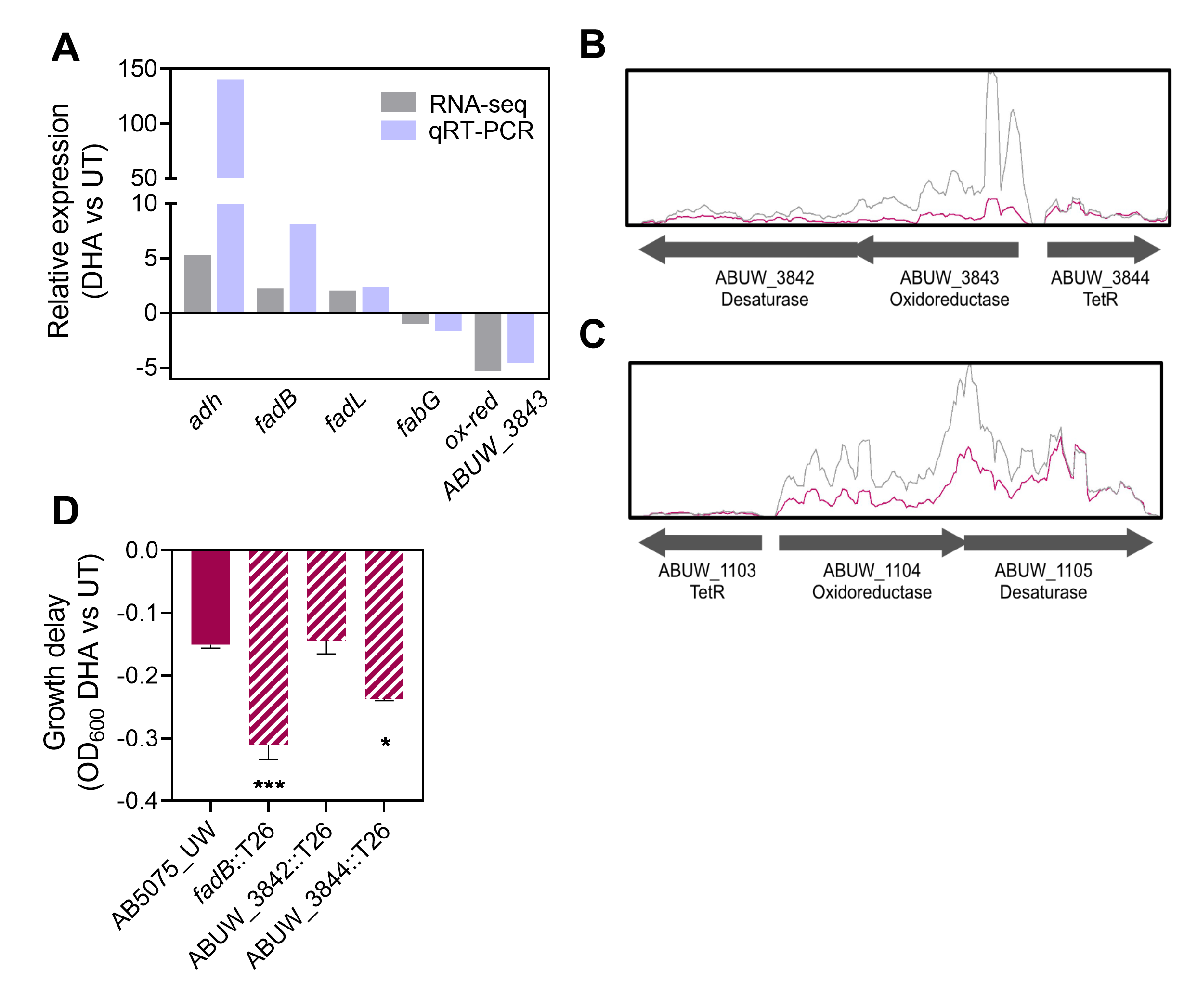

Supplement: FIG S1 [file mbio.01070-21-sf001.tif]

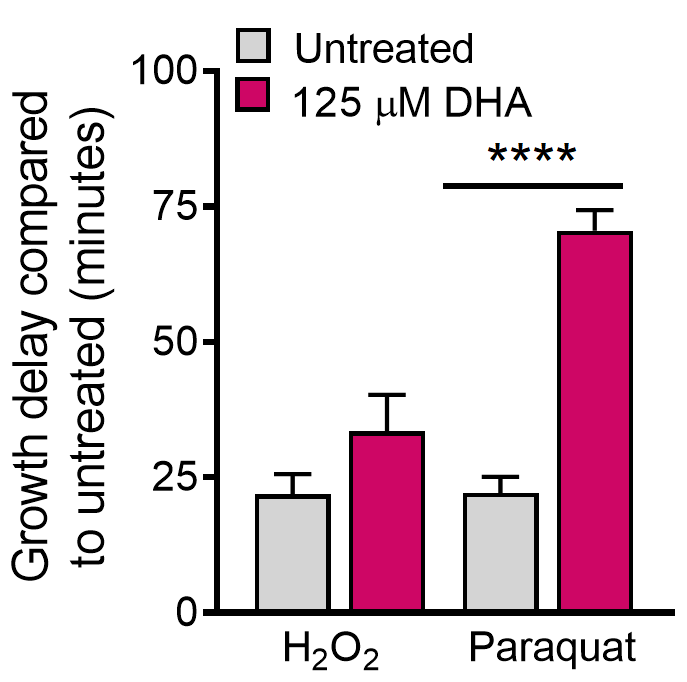

Supplement: FIG S2 [file mbio.01070-21-sf002.tif]
