# Supplementary material for: The Membrane Composition Defines the Spatial Organization and Function of a Major Acinetobacter baumannii Drug Efflux System
Source: mBio. 2021 Jun 17;12(3):e01070-21. doi: 10.1128/mBio.01070-21 (PMC8262998; doi:10.1128/mBio.01070-21)
Supplement: TABLE S1 [file mbio.01070-21-st001.docx]

**Table S1. Transcriptomic responses to DHA stress**

| Locus tag | Fold Change (Log_2_) | Adjusted P value | Product |
| --- | --- | --- | --- |
| ABUW_2767 | 3.54 | 3.3E-36 | monooxygenase |
| ABUW_2540 | 3.19 | 6.2E-01 | transposase |
| ABUW_0802 | 2.86 | 2.6E-01 | hypothetical protein |
| ABUW_1624 | 2.41 | 1.8E-41 | alcohol dehydrogenase, iron-containing |
| ABUW_2768 | 2.39 | 1.1E-13 | short chain dehydrogenase |
| ABUW_0620 | 2.35 | 1.3E-22 | hypothetical protein |
| ABUW_1621 | 2.22 | 7.2E-08 | aldehyde dehydrogenase |
| ABUW_0108 | 2.16 | 2.8E-41 | short-chain dehydrogenase/reductase |
| ABUW_0433 | 2.05 | 1.8E-16 | flavin-containing monooxygenase FMO |
| ABUW_0184 | 2.00 | 5.1E-07 | Na+/solute symporter |
| ABUW_2195 | 1.99 | 8.7E-20 | alkane 1-monooxygenase |
| ABUW_2588 | 1.74 | 3.5E-01 | hypothetical protein |
| ABUW_2009 | 1.74 | 9.6E-01 | phage replication protein |
| ABUW_2784 | 1.72 | 1.0E-01 | hypothetical protein |
| ABUW_2130 | 1.65 | 1.4E-01 | acetoin:2,6-dichlorophenolindophenol oxidoreductase subunit beta |
| ABUW_0183 | 1.64 | 4.2E-05 | hypothetical protein |
| ABUW_0170 | 1.61 | 1.1E-20 | N-ethylmaleimide reductase |
| ABUW_2782 | 1.59 | 2.2E-02 | porin |
| ABUW_2131 | 1.59 | 1.8E-01 | acetoin:2,6-dichlorophenolindophenol oxidoreductase alpha subunit |
| ABUW_0190 | 1.57 | 4.2E-10 | transcriptional regulator, TetR family |
| ABUW_1233 | 1.52 | 2.1E-07 | OmpW family protein |
| ABUW_3307 | 1.51 | 4.6E-08 | enoyl-CoA hydratase |
| ABUW_2595 | 1.49 | 2.3E-07 | hypothetical protein |
| ABUW_0201 | 1.48 | 5.7E-02 | GABA permease |
| ABUW_2783 | 1.48 | 2.0E-01 | hypothetical protein |
| ABUW_0606 | 1.46 | 1.2E-12 | acyl-CoA dehydrogenase |
| ABUW_1006 | 1.45 | 3.8E-10 | enoyl-CoA hydratase/isomerase |
| ABUW_0440 | 1.44 | 7.6E-07 | TRAP C4-dicarboxylate transport system permease |
| ABUW_2129 | 1.43 | 2.1E-01 | dihydrolipoamide acetyltransferase |
| ABUW_2132 | 1.38 | 8.3E-02 | lipoic acid synthetase |
| ABUW_2652 | 1.38 | 6.0E-01 | hypothetical protein |
| ABUW_0324 | 1.38 | 1.5E-06 | lipase |
| ABUW_0169 | 1.38 | 1.2E-12 | transcriptional regulator, ArsR family |
| ABUW_1773 | 1.36 | 2.5E-10 | alpha/beta hydrolase |
| ABUW_3020 | 1.35 | 6.0E-03 | RND family multidrug resistance secretion protein |
| ABUW_2370 | 1.33 | 6.1E-01 | transcriptional regulator, ArsR family |
| ABUW_3291 | 1.31 | 7.6E-07 | acyl-CoA synthase |
| ABUW_3018 | 1.29 | 7.0E-06 | gamma-glutamyltransferase |
| ABUW_2465 | 1.25 | 6.0E-05 | 3-oxoadipate CoA-transferase subunit A |
| ABUW_0439 | 1.25 | 3.3E-06 | rhomboid family peptidase |
| ABUW_2785 | 1.22 | 3.9E-03 | tannase/feruloyl esterase family protein |
| ABUW_1620 | 1.22 | 1.0E-04 | ethanolamine permease |
| ABUW_1836 | 1.22 | 1.8E-04 | 4-hydroxybenzoate transporter |
| ABUW_2128 | 1.22 | 3.0E-01 | dihydrolipoamide dehydrogenase |
| ABUW_0107 | 1.22 | 2.3E-07 | transcriptional regulator, TetR family |
| ABUW_1562 | 1.21 | 4.3E-08 | luciferase family monooxygenase |
| ABUW_2945 | 1.21 | 1.5E-02 | putative hydrolase |
| ABUW_1656 | 1.20 | 9.1E-03 | OmpW family protein |
| ABUW_3396 | 1.19 | 6.0E-04 | phosphate acetyltransferase |
| ABUW_3572 | 1.16 | 3.7E-08 | fatty oxidation complex, alpha subunit FadB |
| ABUW_2041 | 1.15 | 6.9E-01 | hypothetical protein |
| ABUW_1561 | 1.14 | 6.6E-03 | hypothetical protein |
| ABUW_3786 | 1.14 | 7.9E-03 | D-serine/D-alanine/glycine transporter |
| ABUW_3187 | 1.13 | 6.3E-01 | hypothetical protein |
| ABUW_3311 | 1.11 | 1.9E-06 | short chain dehydrogenase |
| ABUW_2781 | 1.10 | 3.8E-02 | long-chain specific acyl-coa dehydrogenase |
| ABUW_2892 | 1.09 | 2.9E-01 | citrate transporter |
| ABUW_1324 | 1.09 | 7.5E-01 | hypothetical protein |
| ABUW_0175 | 1.07 | 1.8E-02 | acetate--CoA ligase |
| ABUW_3245 | 1.06 | 7.0E-04 | FilA |
| ABUW_1463 | 1.06 | 1.8E-04 | omega-amino acid--pyruvate aminotransferase |
| ABUW_3073 | 1.06 | 1.0E-02 | succinate-semialdehyde dehydrogenase (NADP+) |
| ABUW_2604 | 1.05 | 1.2E-01 | allophanate hydrolase |
| ABUW_2965 | 1.05 | 1.3E-01 | hypothetical protein |
| ABUW_3075 | 1.05 | 6.5E-04 | lipolytic enzyme |
| ABUW_1525 | 1.05 | 3.1E-01 | C4-dicarboxylate transport protein |
| ABUW_3149 | 1.04 | 2.9E-01 | hypothetical protein |
| ABUW_3403 | 1.04 | 1.7E-01 | TonB-dependent receptor |
| ABUW_0672 | 1.03 | 4.5E-01 | transposase inhibitor |
| ABUW_2989 | 1.03 | 5.4E-03 | dihydrodipicolinate synthetase |
| ABUW_0724 | 1.03 | 3.7E-04 | FadL outer membrane protein |
| ABUW_2769 | 1.02 | 2.6E-02 | acetyl-hydrolase |
| ABUW_3074 | 1.02 | 1.0E-02 | dioxygenase alpha subunit |
| ABUW_0239 | 1.01 | 1.2E-02 | hypothetical protein |
| ABUW_3807 | 1.00 | 2.9E-02 | 2-methylcitrate synthase |
| ABUW_1206 | 1.00 | 5.4E-03 | dipeptide/tripeptide permease |
| ABUW_2952 | 1.00 | 4.8E-01 | hypothetical protein |
| ABUW_2416 | 1.00 | 8.8E-02 | hypothetical protein |
| ABUW_2127 | 1.00 | 1.1E-02 | acetoin dehydrogenase |
| ABUW_3021 | 1.00 | 2.4E-05 | hypothetical protein |
| ABUW_1277 | -1.00 | 9.8E-01 | hypothetical protein |
| ABUW_1168 | -1.02 | 2.9E-01 | siderophore-interacting protein |
| ABUW_3842 | -1.02 | 3.5E-02 | fatty acid desaturase |
| ABUW_1160 | -1.03 | 8.6E-01 | hypothetical protein |
| ABUW_1498 | -1.03 | 4.7E-01 | transcriptional regulator, TetR family |
| ABUW_2075 | -1.07 | 7.1E-02 | isochorismatase |
| ABUW_0071 | -1.16 | 7.3E-04 | aromatic amino acid transport protein |
| ABUW_2994 | -1.16 | 8.8E-01 | hypothetical protein |
| ABUW_1597 | -1.18 | 1.5E-06 | hypothetical protein |
| ABUW_2076 | -1.20 | 2.6E-04 | 2,3-dihydro-2,3-dihydroxybenzoate dehydrogenase |
| ABUW_1186 | -1.22 | 8.6E-02 | thioesterase |
| ABUW_2056 | -1.27 | 1.8E-03 | hypothetical protein |
| ABUW_1104 | -1.29 | 4.0E-13 | oxidoreductase |
| ABUW_1558 | -1.29 | 2.2E-02 | polysaccharide deacetylase |
| ABUW_1187 | -1.30 | 7.3E-03 | phosphopantetheinyl transferase |
| ABUW_1188 | -1.31 | 6.6E-02 | isochorismate synthetase |
| ABUW_1557 | -1.32 | 2.2E-02 | outer membrane protein |
| ABUW_0069 | -1.32 | 3.6E-02 | maleylacetoacetate isomerase |
| ABUW_2052 | -1.33 | 3.0E-01 | fimbrial subunit |
| ABUW_1559 | -1.33 | 4.5E-02 | glycosyl transferase, family 2 protein |
| ABUW_0260 | -1.34 | 1.8E-04 | acyl coenzyme A reductase |
| ABUW_2178 | -1.39 | 3.2E-05 | rhizobactin siderophore biosynthesis protein RhbD |
| ABUW_1179 | -1.42 | 6.3E-02 | nonribosomal peptide synthetase BasD |
| ABUW_2916 | -1.46 | 9.9E-04 | TonB dependent outer membrane siderophore receptor |
| ABUW_1175 | -1.47 | 4.2E-03 | ferric acinetobactin ATP-binding transporter |
| ABUW_1185 | -1.47 | 5.7E-03 | ABC transporter |
| ABUW_1178 | -1.49 | 5.1E-03 | nonribosomal peptide synthetase BasC |
| ABUW_1184 | -1.50 | 6.6E-03 | ABC transporter, ATP-binding protein |
| ABUW_1170 | -1.52 | 1.3E-04 | non-ribosomal peptide synthetase |
| ABUW_1174 | -1.53 | 6.0E-03 | ferric acinetobactin transport system permease |
| ABUW_1182 | -1.57 | 4.8E-02 | histidine decarboxylase |
| ABUW_1176 | -1.61 | 1.3E-04 | ferric acinetobactin transport system periplasmic protein |
| ABUW_0068 | -1.65 | 1.6E-02 | glyoxalase/bleomycin resistance protein/dioxygenase |
| ABUW_1177 | -1.69 | 1.1E-05 | ferric acinetobactin receptor |
| ABUW_3126 | -1.71 | 8.4E-01 | hypothetical protein |
| ABUW_1181 | -1.72 | 5.8E-02 | 2,3 dihydro-2,3 dihydroxybenzoate synthase |
| ABUW_1180 | -1.74 | 1.8E-02 | 2,3-dihydroxybenzoate-AMP ligase |
| ABUW_1307 | -1.91 | 9.5E-01 | hypothetical protein |
| ABUW_2227 | -1.91 | 9.5E-01 | hypothetical protein |
| ABUW_1172 | -2.00 | 6.1E-03 | hypothetical protein |
| ABUW_1183 | -2.08 | 1.8E-03 | hypothetical protein |
| ABUW_1173 | -2.09 | 2.2E-09 | ferric acinetobactin transport system permease |
| ABUW_1171 | -2.15 | 3.6E-05 | hypothetical protein |
| ABUW_1352 | -2.23 | 1.7E-12 | hypothetical protein |
| ABUW_0259 | -2.27 | 8.1E-05 | sulfate transporter |
| ABUW_3843 | -2.40 | 1.4E-10 | putative oxidoreductase |
| ABUW_0757 | -2.74 | 8.6E-01 | hypothetical protein |
