# Supplementary material for: The Membrane Composition Defines the Spatial Organization and Function of a Major Acinetobacter baumannii Drug Efflux System
Source: mBio. 2021 Jun 17;12(3):e01070-21. doi: 10.1128/mBio.01070-21 (PMC8262998; doi:10.1128/mBio.01070-21)
Supplement: TABLE S2 [file mbio.01070-21-st002.docx]

**Table S2. Minimal inhibitory concentration of *Streptococcus pneumoniae* with and without DHA treatment**

|  | *S. pneumoniae* D39 (µg.ml^-1^)*^a^* | *S. pneumoniae* D39 + 64 µM DHA (µg.ml^-1^)*^a^* |
| --- | --- | --- |
| Erythromycin | 0.06 | 0.03 |
| Azithromycin | 0.25 | 0.125 |
| Gentamicin | 8 | 4 |
| Streptomycin | 16 | 8 |
| Chloramphenicol | 2 | 2 |

*^a^*Data represent the mode of 3 biological replicates.
