# Supplementary material for: The Membrane Composition Defines the Spatial Organization and Function of a Major Acinetobacter baumannii Drug Efflux System
Source: mBio. 2021 Jun 17;12(3):e01070-21. doi: 10.1128/mBio.01070-21 (PMC8262998; doi:10.1128/mBio.01070-21)
Supplement: TABLE S3 [file mbio.01070-21-st003.docx]

**Table S3.** **Electrochemical properties of tethered bilayer lipid membranes**

| Sample | Electrolyte | Membrane resistance (MOhm.cm^2^) | Membrane capacitance (µF.cm^2^) | alpha |
| --- | --- | --- | --- | --- |
| Bilayer (no DHA) | 10 mM PBS | 52.9 ± 8% | 0.089 ± 3% | 0.98 |
| + valinomycin | 100 mM NaCl | 26.6 ± 6% | 0.093 ± 2% | 0.98 |
|  | 100 mM KCl | 0.527 ± 6% | 1.2 ± 4% | 0.93 |
|  | 100 mM NaCl | 31.5 ± 6% | 0.88 ± 2% | 0.98 |
| Bilayer (with DHA) | 10 mM PBS | 50.1 ± 10% | 1.0 ± 4% | 0.96 |
| + valinomycin | 100 mM NaCl | 31.5 ± 7% | 1.1 ± 2% | 0.95 |
|  | 100 mM KCl | 0.806 ± 10% | 1.5 ± 4% | 0.96 |
|  | 100 mM NaCl | 18.8 ± 15% | 1.2 ± 6% | 0.96 |
