# Supplementary material for: The Membrane Composition Defines the Spatial Organization and Function of a Major Acinetobacter baumannii Drug Efflux System
Source: mBio. 2021 Jun 17;12(3):e01070-21. doi: 10.1128/mBio.01070-21 (PMC8262998; doi:10.1128/mBio.01070-21)
Supplement: TEXT S1 [file mbio.01070-21-s0001.docx]

**Supplementary Methods**

***Strains and culture media***

*A. baumannii* strains (**Table S4**) were routinely grown in Luria Bertani broth (LB) or LB agar (1.5%). The *fadL*, *adeJ* and *adeB* AB5075_UW transposon mutants were purchased from the Manoil Laboratory (University of Washington). The *A. baumannii* ATCC 17978 ∆*fadL* strain as part of this study, was generated using an established strategy (1, 2) (oligonucleotides are listed in **Table S5**) and confirmed by whole genome sequencing (see methodology below). The opaque state for all AB5075_UW derivatives was confirmed by oblique light microscopy (3).

***Bacterial growth assays***

Cultures in LB media were incubated at 37°C with shaking in a FLUOStar Omega Spectrophotometer (BMG Labtech), with the mean OD_600_ values determined every 15 minutes. The 20 mL cultures used for all other analyses were incubated at 37°C in an Innova 40R shaking incubator (Eppendorf) at 230 rpm until they reached mid log-phase (OD_600_ = 0.7).

***Lipid extraction***

Overnight cultures of *A. baumannii* AB5075_UW were diluted to an OD_600_ of 0.05 in fresh LB media (20 ml) and grown to mid-log phase (OD_600_ = 0.7), treated cultures were supplemented with 0.25 mM DHA. Cells were harvested by centrifugation at 7,000 × *g* for 10 minutes and washed once with PBS, processed pellets were then resuspended in 50 μl of 1.5% NaCl  buffer. For lipid extraction, 1 ml of chloroform:methanol (2:1; v/v) was added to the cell suspension, mixed vigorously for 2 minutes then incubated at RT for 10 minutes. Following the addition of 200 μl 1.5% NaCl, the cell suspension was mixed vigorously for 1 minute and centrifuged at 6,000 × *g* for phase-separation. The lower phase was recovered and concentrated via nitrogen evaporation.

***tBLM formation and characterisation***

Tethered bilayer lipid membranes were formed and analysed using established protocols (4, 5). Lipid extracts from *A. baumannii* grown with and without DHA were extruded to form 200 nm vesicles, which were fused with a pre-assembled fully tethered DPhyTL monolayer. The formation of a bilayer was monitored using electrochemical impedance spectroscopy (EIS) using a Metrohm PGSTAT 12 spectrometer, with a membrane coated gold working electrode, a platinum counter and a Ag/AgCl reference electrode. The electrochemical properties of the membrane were modelled using an equivalent circuit of resistors and capacitors, as described elsewhere (4, 5). Functionality of the membrane was shown by incorporation of valinomycin and monitoring ion transport. tBLMs were analysed by EIS and modelled to an R(RCPE) equivalent circuit, where the bilayer is described as a parallel arrangement of a resistor and a capacitor (6). A constant phase element was used instead of a capacitor, to accommodate for small inhomogeneities in the membrane, the alpha parameter being proportional to the distribution of capacitors. An alpha value of 1 would correspond to a pure capacitor.

***Transcriptomic analyses***

Three independent *A. baumannii* AB5075_UW cultures were grown to mid-log phase (OD_600_ = 0.6). Each culture was divided into two culture flasks, one treated with 250 µM DHA (Sigma-Aldrich) and the other left untreated as a control and both incubated with shaking for 1 hour at 37^o^C prior to cell harvest. RNA extraction was carried out using the miRNeasy mini kit (Qiagen) and DNA was eliminated using the TURBO DNA-free kit (Ambion Inc, USA), as per manufacturer’s instructions. The cDNA library was generated using the TruSeq^®^ Stranded Total RNA Sample Preparation kit (Illumina, Inc., USA). The samples were sequenced on an Illumina HiSeq4000 platform yielding approximately 20 million 100 bp paired-end reads per sample. The raw sequencing data has been submitted to the Gene Expression Omnibus (accession number GSE131943). The reads were analysed using EDGE-pro (Estimated Degree of Gene Expression in Prokaryotic Genomes) (7). Reads were aligned against the *A. baumannii* AB5075_UW reference genome (Genbank accession: CP008706.1). Genes with differential expression were identified using the R package DESeq2 (8) (**Table S1**).

***Minimal Inhibitory Concentration assays***

The antimicrobial resistance profile of *A. baumannii* AB5075_UW was assessed using the micro-dilution method in cation-adjusted Mueller Hinton broth as previously described (9). To assess the impact of PUFAs, media was supplemented with 250 μM DHA. Plates were sealed with a breathable film, placed in a humidity box and incubated overnight at 37^o^C. Following incubation, MIC values were determined by visual examination of growth inhibition.

***DNA extraction and whole genome sequencing***

DNA was extracted on a QIASymphony SP with QIASymphony DSP Virus/Pathogen kit (QIAGEN, Hilden, Germany) as per the manufacturer’s instructions. The DNA concentration was quantified using a Quant-IT dsDNA High Sensitivity kit (ThermoFisher Scientific, MA, USA).

Sequencing libraries were prepared from the pure *A. baumannii* DNA extracts sample using Illumina Nextera XT Library Preparation kit (Illumina Inc., CA, USA) with slight modifications. Half volume was used for tagmentation reagents, amplification reagents and input DNA. Library clean-up was performed using AxyPrep MAG PCR Clean up kit (Corning Inc., NY, USA), and libraries were pooled manually, and sequenced on a NextSeq 550 platform with NextSeq 500/550 Mid-Output kit v2.5 (300 cycles) (Illumina Inc.).

***Ethidium Bromide accumulation***

Cultures were grown to mid-log phase (OD_600_ = 0.7) with or without 250 μM DHA. Washed cells were incubated with or without 40 μM of the protonophore carbonyl cyanide-*m*-chlorophenyl hydrazone (CCCP, Sigma Aldrich) for 10 minutes. The cells were then supplemented with 5 μM of ethidium bromide (EtBr) (Sigma Aldrich). The fluorescence intensity (excitation/emission = 530/600 nm) was measured using a CLARIOstar Plus Microplate Reader (BMG Labtech).

***Computational Methodology***

To examine the effect of PUFA incorporation on AdeJ and AdeB, two model membranes were constructed based on lipidomic profiling performed by Jiang *et al.* using the MARTINI coarse-grain lipid forcefield (**Table S6**) (10-12). The two membranes represented no PUFA treatment or treatment with DHA respectively. Go-MARTINI (13) models of AdeB and AdeJ (both based on the structure of AdeB (14)) were embedded in the untreated and DHA treated model membranes. Replicate 15 μs simulations (n = 3) of each system were run using the GROMACS 2019.4 simulation engine (15, 16) prior to analysis.

***Membrane composition data analysis***

For each of the two membrane conditions (untreated, and DHA treated), gas chromatography (GC) and liquid chromatography-mass spectrometry (LC-MS) data from Jiang *et al* (10) provided the relative proportion of lipid head groups phosphatidylethanolamine (PE), phosphatidylglycerol (PG) and cardiolipin (CL), the relative total abundance of various tail species and the abundance of various headgroup-tail combinations. This data is not resolved between the inner and outer membrane or between leaflets therein. As such, the resulting bilayer compositions should be viewed as simplified model systems.

The large pool of phospholipid species was streamlined by removing any individual species with < 5% abundance within a specific headgroup pool (e.g. < 5% of the total PE pool). Total abundances were then rounded to a whole number to obtain a membrane composition that could be used for simulation (**Table S6**). As LC-MS data did not distinguish individual lipid tails and provided only total number of carbons and number of unsaturated positions, individual species retained in the streamlined pool were then assigned specific lipid tails based on the GC data. Further, in assigning phospholipid tails, common tail combinations were preferred, where these could not be resolved on the basis of LC-MS and GC data (17-19). The majority of CL species were assigned palmitic and oleic tails, in agreement with results from a recent mass-spectrometry study (20).

***Coarse grained mapping and parameter determination***

Following determination of individual lipid species abundance, each species was mapped to its coarse-grained (CG) representation using the MARTINI forcefield (11, 21). Given the lower resolution of CG simulations, lipid species sometimes map onto the same set of CG beads. Thus, following determination of unique CG representations, relative abundances were updated to reflect lipids mapping to the same set of beads (**Table S6**). Most coarse-grain lipid types were already listed on the MARTINI database with their corresponding itp file, while some were not. For the latter group, itp files were created by hand or with MARTINI lipid making software (13). Parameters for new lipids were constructed according to the standard MARTINI 2 lipid building blocks. CL parameters are those of Dahlberg (22, 23). Following experimental observations of cardiolipin protonation states at physiological pH, we assigned cardiolipins to be deprotonated (di-anionic). This was reflected in their itp files (24).

***Homology model construction***

A homology model of AdeJ in the resting state, based on the resting state AdeB cryo-EM structure (14) (PDB ID: 6OWS, 44.5% sequence identity), was developed using the protein fold recognition approach implemented in the Phyre2 webserver (25). Each subunit of AdeJ was modelled on the corresponding subunit of AdeB.

***System preparation***

Membranes were constructed using a custom version of the *insane* (26) tool for CG bilayer creation. In the absence of any information on leaflet asymmetry, leaflets were assumed to have identical compositions. AdeB and AdeJ were coarse-grained using the Go-MARTINI approach (13). AdeB and AdeJ were embedded in the untreated and DHA treated membranes oriented in the x-y plane of a solvated rectangular box with dimensions of 30 nm x 30 nm x 21 nm. Each membrane contained ~3000 lipids. Due to the known limitations with the standard Martini coarse-grain water, the system was solvated using the polarizable MARTINI water model (27), with 150 mM sodium chloride. Each system contained ~390,000 CG beads.

All simulations were run using GROMACS 2019.4 (15, 16, 28) under periodic boundary conditions and employed standard MARTINI2 simulation settings (29). The dielectric constant was set to 2.5 for use with the MARTINI polarizable water model. The temperature was maintained at 310 K using the Bussi thermostat (30) and a coupling constant of 1.0 ps. During equilibration, a semi-isotropic Berendsen pressure coupling (31) was used to maintain the pressure at 1 bar using a coupling constant of 5 ps. Production simulations used a semi-isotropic Parinello-Rahaman barostat (32, 33) to maintain a pressure of 1 bar with a compressibility of 3 × 10^-4^ bar^-1^ and a coupling constant of 12 ps. All relevant force field files, and input coordinates can be found at <https://github.com/hmacdope/Abaumanii_MARTINI_membrane>.

***Equilibration***

The membrane-embedded AdeJ and AdeB systems were equilibrated using a series of five 2.5 ns simulations in which the position restraints on the protein were sequentially relaxed from 1000 kJ/mol/nm^2^ to 100 kJ/mol/nm^2^, 50 kJ/mol/nm^2^, 10 kJ/mol/nm^2^ and finally 0 kJ/mol/nm^2^ using a 5 fs time step. Throughout these simulations, weak z-axis position restraints (force constant of 2 kJ/mol/nm^2^) were applied to the PO4 bead of each phospholipid to limit large scale bilayer undulations. Each system was equilibrated for a further 5 ns in unrestrained equilibrations with a timestep of 10 fs (for 5 ns) and further 7.5 ns using a 15 fs timestep.

***Production simulation***

For the membrane-embedded AdeJ and AdeB systems, three replicate 15 μs simulations were conducted. No z-axis restraints on the phospholipid headgroups were used. We note that simulation timescales reported in this work are exact, and do not apply the four-fold multiplier to account for enhanced diffusion rate as is sometimes used in the literature.

**References**

1. Adams FG, Stroeher UH, Hassan KA, Marri S, Brown MH. 2018. Resistance to pentamidine is mediated by AdeAB, regulated by AdeRS, and influenced by growth conditions in *Acinetobacter baumannii* ATCC 17978. PLoS One 13:e0197412.

2. Tucker AT, Nowicki EM, Boll JM, Knauf GA, Burdis NC, Trent MS, Davies BW. 2014. Defining gene-phenotype relationships in *Acinetobacter baumannii* through one-step chromosomal gene inactivation. mBio 5:e01313-14.

3. Tipton KA, Dimitrova D, Rather PN. 2015. Phase-Variable Control of Multiple Phenotypes in Acinetobacter baumannii Strain AB5075. J Bacteriol 197:2593-9.

4. Vockenroth IK, Rossi C, Shah MR, Köper I. 2009. Formation of tethered bilayer lipid membranes probed by various surface sensitive techniques. Biointerphases 4:19-26.

5. Köper I. 2007. Insulating tethered bilayer lipid membranes to study membrane proteins. Molecular BioSystems 3:651-657.

6. Andersson J, Köper I. 2016. Tethered and polymer supported bilayer lipid membranes: structure and function. Membranes 6:30.

7. Magoc T, Wood D, Salzberg SL. 2013. EDGE-pro: Estimated Degree of Gene Expression in prokaryotic genomes. Evol Bioinform Online 9:127-36.

8. Love MI, Huber W, Anders S. 2014. Moderated estimation of fold change and dispersion for RNA-seq data with DESeq2. Genome Biol 15:550.

9. Wiegand I, Hilpert K, Hancock REW. 2008. Agar and broth dilution methods to determine the minimal inhibitory concentration (MIC) of antimicrobial substances. Nature Protocols 3:163-175.

10. Jiang JH, Hassan KA, Begg SL, Rupasinghe TWT, Naidu V, Pederick VG, Khorvash M, Whittall JJ, Paton JC, Paulsen IT, McDevitt CA, Peleg AY, Eijkelkamp BA. 2019. Identification of novel *Acinetobacter baumannii* host fatty acid stress adaptation strategies. MBio 10:  e02056-18.

11. Marrink SJ, De Vries AH, Mark AE. 2004. Coarse grained model for semiquantitative lipid simulations. Journal of Physical Chemistry B 108:750-760.

12. Marrink SJ, Risselada HJ, Yefimov S, Tieleman DP, De Vries AH. 2007. The MARTINI force field: Coarse grained model for biomolecular simulations. Journal of Physical Chemistry B 111:7812-7824.

13. Poma AB, Cieplak M, Theodorakis PE. 2017. Combining the MARTINI and structure-based coarse-grained approaches for the molecular dynamics studies of conformational transitions in proteins. Journal of Chemical Theory and Computation 13:1366-1374.

14. Su C-C, Morgan CE, Kambakam S, Rajavel M, Scott H, Huang W, Emerson CC, Taylor DJ, Stewart PL, Bonomo RA, Yu EW. 2019. Cryo-electron microscopy structure of an *Acinetobacter baumannii* multidrug efflux pump. mBio 10:e01295-19.

15. Abraham MJ, Murtola T, Schulz R, Páll S, Smith JC, Hess B, Lindahl E. 2015. GROMACS: High performance molecular simulations through multi-level parallelism from laptops to supercomputers. SoftwareX 1-2:19-25.

16. Van Der Spoel D, Lindahl E, Hess B, Groenhof G, Mark AE, Berendsen HJ. 2005. GROMACS: fast, flexible, and free. J Comput Chem 26:1701-18.

17. Im W, Khalid S. 2020. Molecular simulations of Gram-negative bacterial membranes come of age. Annual Review of Physical Chemistry 71:171-188.

18. Sohlenkamp C, Geiger O. 2015. Bacterial membrane lipids: Diversity in structures and pathways, vol 40, p 133-159. Oxford University Press.

19. Appala K, Bimpeh K, Freeman C, Hines KM. 2020. Recent applications of mass spectrometry in bacterial lipidomics. Analytical and Bioanalytical Chemistry doi:10.1007/s00216-020-02541-8.

20. Lopalco P, Stahl J, Annese C, Averhoff B, Corcelli A. 2017. Identification of unique cardiolipin and monolysocardiolipin species in *Acinetobacter baumannii*. Scientific Reports 7:2972.

21. Marrink SJ, Risselada HJ, Yefimov S, Tieleman DP, de Vries AH. 2007. The MARTINI force field: coarse grained model for biomolecular simulations. J Phys Chem B 111:7812-24.

22. Dahlberg M. 2007. Polymorphic phase behavior of cardiolipin derivatives studied by coarse-grained molecular dynamics. The Journal of Physical Chemistry B 111:7194-7200.

23. Dahlberg M, Maliniak A. 2010. Mechanical properties of coarse-grained bilayers formed by cardiolipin and zwitterionic lipids. Journal of Chemical Theory and Computation 6:1638-1649.

24. Sathappa M, Alder NN. 2016. The ionization properties of cardiolipin and its variants in model bilayers. Biochimica et Biophysica Acta (BBA) - Biomembranes 1858:1362-1372.

25. Kelley LA, Mezulis S, Yates CM, Wass MN, Sternberg MJ. 2015. The Phyre2 web portal for protein modeling, prediction and analysis. Nat Protoc 10:845-58.

26. Wassenaar TA, Ingólfsson HI, Böckmann RA, Tieleman DP, Marrink SJ. 2015. Computational lipidomics with insane: A versatile tool for generating custom membranes for molecular simulations. Journal of Chemical Theory and Computation 11:2144-2155.

27. Yesylevskyy SO, Schäfer LV, Sengupta D, Marrink SJ. 2010. Polarizable water model for the coarse-grained MARTINI force field. PLOS Computational Biology 6:e1000810.

28. Berendsen HJC, van der Spoel D, van Drunen R. 1995. GROMACS: A message-passing parallel molecular dynamics implementation. Computer Physics Communications 91:43-56.

29. de Jong DH, Baoukina S, Ingólfsson HI, Marrink SJ. 2016. Martini straight: Boosting performance using a shorter cutoff and GPUs. Computer Physics Communications 199:1-7.

30. Bussi G, Donadio D, Parrinello M. 2007. Canonical sampling through velocity rescaling. The Journal of Chemical Physics 126:014101.

31. Berendsen HJC, Postma JPM, van Gunsteren WF, DiNola A, Haak JR. 1984. Molecular dynamics with coupling to an external bath. The Journal of Chemical Physics 81:3684-3690.

32. Parrinello M, Rahman A. 1981. Polymorphic transitions in single crystals: A new molecular dynamics method. Journal of Applied Physics 52:7182-7190.

33. Parrinello M, Rahman A. 1980. Crystal structure and pair potentials: A molecular-dynamics study. Physical Review Letters 45:1196-1199.
