# Supplementary material for: The Membrane Composition Defines the Spatial Organization and Function of a Major Acinetobacter baumannii Drug Efflux System
Source: mBio. 2021 Jun 17;12(3):e01070-21. doi: 10.1128/mBio.01070-21 (PMC8262998; doi:10.1128/mBio.01070-21)
Supplement: TABLE S4 [file mbio.01070-21-st004.docx]

**Table S4. Bacterial strains included in the study**

| Strain | Genotype or description*^a^* | Reference/ source*^b^* |
| --- | --- | --- |
| *A. baumannii* AB5075_UW | Wild-type | Manoil Lab (1) |
| *adeB*::T26 | AB5075_UW with transposon (T26) disrupted *adeB* (ABUW_1975)*,* Tet^R^ | Manoil Lab (1) |
| *adeJ*::T26 | AB5075_UW with transposon (T26) disrupted *adeJ* (ABUW_0843)*,* Tet^R^ | Manoil Lab (1) |
| *fadL*::T26 | AB5075_UW with transposon (T26) disrupted *fadL* (ABUW_0724)*,* Tet^R^ | Manoil Lab (1) |
| *fadB*::T26 | AB5075_UW with transposon (T26) disrupted *fadB* (ABUW_3572)*,* Tet^R^ | Manoil Lab (1) |
| ABUW_3842::T26 | AB5075_UW with transposon (T26) disrupted ABUW_3842*,* Tet^R^ | Manoil Lab (1) |
| ABUW_3844::T26 | AB5075_UW with transposon (T26) disrupted ABUW_3844*,* Tet^R^ | Manoil Lab (1) |
| ATCC 17978 | Wild-type | ATCC |
| ATCC 17978 + pAT04 | ATCC 17978 carrying IPTG-inducible recombinase plasmid pAT04 | Davies Lab (2) |
| ∆*fadL* | ATCC 17978 with Kan^R^ insertion disruption in *fadL* (A1S_2773) | This study |

*^a^* Kan^R^, kanamycin resistant; Tet^R^, tetracycline resistant. *^b^* **(1)** Gallagher LA, Ramage E, Weiss EJ, Radey M, Hayden HS, Held KG, Huse HK, Zurawski DV, Brittnacher MJ, Manoil C. 2015. Resources for genetic and genomic analysis of emerging pathogen *Acinetobacter baumannii*. Journal of Bacteriology 197:2027. **(2)** Tucker AT, Nowicki EM, Boll JM, Knauf GA, Burdis NC, Trent MS, Davies BW. 2014. Defining gene-phenotype relationships in *Acinetobacter baumannii* through one-step chromosomal gene inactivation. mBio 5:e01313-14.
