# Supplementary material for: The Membrane Composition Defines the Spatial Organization and Function of a Major Acinetobacter baumannii Drug Efflux System
Source: mBio. 2021 Jun 17;12(3):e01070-21. doi: 10.1128/mBio.01070-21 (PMC8262998; doi:10.1128/mBio.01070-21)
Supplement: TABLE S5 [file mbio.01070-21-st005.docx]

**Table S5. Oligonucleotides included in the study**

| Name | Forward (5’ -> 3’) | Reverse (5’ -> 3’) |
| --- | --- | --- |
| *fadL*_UFR | CCTGATTGTTCATGCTCATC | CAGCTCCAGCCTACACAATCGCATCATTGCAGTGCTCAGATG |
| *fadL*_DFR | AAGGAGGATATTCATATGGACCAATGCTATCGCTTACGG | CATGGCTCACAACCAATCGA |
| *fadL*_NOL | CGACAGCACAGCAAACCT | ACGCTCGTGTAGAGGGTT |
| Kan | GCGATTGTGTAGGCTGGAGCTG | GTCCATATGAATATCCTCCTT |
| qPCR_*GAPDH* | CACCGTCGTACACGTGTTGT | CAACATCACCGCCTTTTTCT |
| qPCR_*adh* | GCACACGCATCAGTAATTGG | CTCACGTAAAAATGGCTATT |
| qPCR_*fadB* | TCGCGGCTGTAAAAGCTAAT | CTATTGAAGATTTCGTGAAC |
| qPCR_*fadL* | CAATCGCAGTGGCTGATAAA | ATGGCGAATGACTACTACTT |
| qPCR_*fabG* | AACAAGGTGCAGGTTTGGCT | ATCCCAATCATCTTCCGACA |
| qPCR_*ABUW_3843* | TGCTGTTTCAAAGCTTGCTG | TCTCGTGCAGCTCAACTATT |
| Kan | GCGATTGTGTAGGCTGGAGCTG | GTCCATATGAATATCCTCCTT |
