# Supplementary material for: The Membrane Composition Defines the Spatial Organization and Function of a Major Acinetobacter baumannii Drug Efflux System
Source: mBio. 2021 Jun 17;12(3):e01070-21. doi: 10.1128/mBio.01070-21 (PMC8262998; doi:10.1128/mBio.01070-21)
Supplement: TABLE S6 [file mbio.01070-21-st006.docx]

| Lipid Headgroup^*^ | Tails^*^ | Simplified %^*^ | Tail 1 (sn-2)^†^ | Tail 2 (sn-1)^†^ | Tail 3 (sn-2)^†^ | Tail 4 (sn-1)^†^ | Martini Lipid Designation  (sn-1, sn-2, sn1-sn2) ^†^ | | Final %^†^ | | Lipid Class |
| --- | --- | --- | --- | --- | --- | --- | --- | --- | --- | --- | --- |
| Untreated *^a^* | | | | | | | | | | | |
| PG | 34:0 | 2 | 16:0 | 18:0 | - | - | DPPG | 2 | | SFA PG | |
| PG | 34:1 | 22 | 18:1 | 16:0 | - | - | POPG | 22 | | UFA PG | |
| PG | 34:2 | 10 | 16:1 | 18:1 | - | - | DOPG | 10 | | UFA PG | |
| PG | 36:3 | 12 | 18:2 | 18:1 | - | - | OIPG | 12 | | UFA PG | |
| CL | 68:1 | 7 | 18:1 | 16:0 | 18:0 | 16:0 | POPP-CL | 10 | | UFA CL | |
| CL | 70:1 | 3 | 18:1 | 16:0 | 18:0 | 18:0 | POPP-CL |  |  |  |  |
| CL | 68:0 | 8 | 16:0 | 18:0 | 16:0 | 18:0 | PPPP-CL | 8 | | SFA CL | |
| CL | 68:2 | 5 | 18:1 | 16:0 | 18:1 | 16:0 | POPO-CL | 5 | | UFA CL | |
| CL | 75:5 | 3 | 18:2 | 18:1 | 18:1 | 21:1 | OIGO-CL | 3 | | UFA CL | |
| CL | 75:6 | 3 | 18:2 | 18:3 | 18:1 | 21:1 | FIGO-CL | 3 | | UFA CL | |
| CL | 75:7 | 4 | 18:3 | 20:1 | 17:0 | 20:1 | GFGP-CL | 4 | | UFA CL | |
| PE | 34:1 | 10 | 18:1 | 16:0 | - | - | POPE | 11 | | UFA PE | |
| PE | 36:1 | 1 | 18:1 | 18:0 | - | - | POPE |  |  |  |  |
| PE | 34:2 | 6 | 16:1 | 18:1 | - | - | DOPE | 10 | | UFA PE | |
| PE | 36:2 | 4 | 18:1 | 18:1 | - | - | DOPE |  |  |  |  |
| AA treated *^a^* | | | | | | | | | | | |
| PG | 34:0 | 6 | 16:0 | 18:0 | - | - | DPPG | 6 | | SFA PG | |
| PG | 34:1 | 12 | 18:1 | 18:0 | - | - | POPG | 12 | | UFA PG | |
| PG | 34:2 | 3 | 16:1 | 18:1 | - | - | DOPG | 3 | | UFA PG | |
| PG | 36:5 | 24 | 20:4 | 16:1 | - | - | OAPG | 27 | | PUFA PG | |
| PG | 38:5 | 3 | 20:4 | 18:1 | - | - | OAPG |  |  |  |  |
| CL | 66:1 | 3 | 18:1 | 16:0 | 16:0 | 16:0 | POPP-CL | 13 | | UFA CL | |
| CL | 67:1 | 6 | 18:1 | 17:0 | 16:0 | 18:0 | POPP-CL |  |  |  |  |
| CL | 68:1 | 4 | 18:1 | 16:0 | 18:0 | 16:0 | POPP-CL |  |  |  |  |
| CL | 68:0 | 4 | 16:0 | 18:0 | 16:0 | 18:0 | PPPP-CL | 4 | | SFA CL | |
| CL | 68:3 | 4 | 18:3 | 16:0 | 18:0 | 16:0 | PFPP-CL | 4 | | UFA CL | |
| CL | 70:4 | 7 | 20:4 | 18:0 | 16:0 | 16:0 | PAPP-CL | 7 | | PUFA CL | |
| PE | 34:1 | 11 | 18:1 | 16:0 | - | - | POPE | 11 | | UFA PE | |
| PE | 36:4 | 13 | 20:4 | 16:0 | - | - | PAPE | 13 | | PUFA PE | |
| DHA treated *^a^* | | | | | | | | | | | |
| PG | 32:0 | 6 | 16:0 | 16:0 | - | - | DPPG | 6 | | SFA PG | |
| PG | 32:1 | 4 | 16:1 | 16:0 | - | - | POPG | 22 | | UFA PG | |
| PG | 34:1 | 18 | 18:1 | 16:0 | - | - | POPG |  |  |  |  |
| PG | 34:2 | 4 | 16:1 | 18:1 | - | - | DOPG | 4 | | UFA PG | |
| PG | 36:3 | 3 | 18:2 | 18:1 | - | - | OIPG | 3 | | UFA PG | |
| PG | 36:6 | 14 | 20:5 | 16:1 | - | - | OUPG | 14 | | PUFA PG | |
| PG | 38:6 | 3 | 22:6 | 16:0 | - | - | PUPG | 3 | | PUFA PG | |
| CL | 66:1 | 4 | 18:1 | 16:0 | 16:0 | 16:0 | POPP-CL | 13 | | UFA CL | |
| CL | 68:1 | 6 | 18:1 | 16:0 | 18:0 | 16:0 | POPP-CL |  |  |  |  |
| CL | 70:1 | 3 | 18:1 | 16:0 | 18:0 | 18:0 | POPP-CL |  |  |  |  |
| CL | 68:0 | 7 | 16:0 | 18:0 | 16:0 | 18:0 | PPPP-CL | 7 | | SFA CL | |
| CL | 70:5 | 4 | 20:5 | 16:0 | 18:0 | 16:0 | PUPP-CL | 6 | | PUFA CL | |
| CL | 75:5 | 2 | 20:5 | 18:0 | 18:0 | 16:0 | PUPP-CL |  |  |  |  |
| PE | 34:1 | 11 | 18:1 | 16:0 | - | - | POPE | 11 | | UFA PE | |
| PE | 34:2 | 2 | 18:1 | 16:1 | - | - | DOPE | 2 | | UFA PE | |
| PE | 36:5 | 8 | 20:5 | 16:0 | - | - | PUPE | 8 | | PUFA PE | |
| PE | 36:6 | 1 | 20:5 | 16:1 | - | - | OUPE | 1 | | PUFA PE | |

**Table S6. Phospholipid species included in membrane modelling**

*^a^* Streamlined lipidomic data and composition of the membranes used in this work. The superscript ^*^ indicates experimental data while the superscript ^†^ indicates parameters derived from experimental data used in the CG membrane models. We note that there is no naming convention for cardiolipins in MARTINI, instead the tails on each CL are indicated with their one letter code (e.g. PPPP-CL corresponds to tetra-palmitoyl-cardiolipin).
